# Identification of mutants with increased variation in cell size at onset of mitosis in fission yeast

**DOI:** 10.1242/jcs.251769

**Published:** 2021-02-11

**Authors:** Elizabeth Scotchman, Kazunori Kume, Francisco J. Navarro, Paul Nurse

**Affiliations:** 1 Cell Cycle Laboratory, The Francis Crick Institute, London NW1 1AT, UK; 2 Graduate School of Integrated Sciences for Life, Hiroshima University, Higashi-Hiroshima, Hiroshima 739-8530, Japan; 3 Hiroshima Research Center for Healthy Aging (HiHA), Hiroshima University,Higashi-Hiroshima, Hiroshima 739-8530, Japan; 4 Laboratory of Yeast Genetics and Cell Biology, Rockefeller University, New York, NY 10065, USA

**Keywords:** Fission yeast, Mitosis, Size control, *mga2*

## Abstract

Fission yeast cells divide at a similar cell length with little variation about the mean. This is thought to be the result of a control mechanism that senses size and corrects for any deviations by advancing or delaying onset of mitosis. Gene deletions that advance cells into mitosis at a smaller size or delay cells entering mitosis have led to the identification of genes potentially involved in this mechanism. However, the molecular basis of this control is still not understood. In this work, we have screened for genes that when deleted increase the variability in size of dividing cells. The strongest candidate identified in this screen was *mga2*. The *mga2* deletion strain shows a greater variation in cell length at division, with a coefficient of variation (CV) of 15–24%, while the wild-type strain has a CV of 5–8%. Furthermore, unlike wild-type cells, the *mga2* deletion cells are unable to correct cell size deviations within one cell cycle. We show that the *mga2* gene genetically interacts with *nem1* and influences the nuclear membrane and the nuclear–cytoplasmic transport of CDK regulators.

## Abstract (precis)

**Highlighted Article:** Fission yeast mutants with increased size variation at cell division identify new underlying processes involved in the cell size control mechanism

## INTRODUCTION

Steady-state exponentially growing eukaryotic cells tend to divide with a similar size. This is thought to come about by a size control mechanism acting over the progression of the cell cycle, which sets cell size at mitosis and the subsequent cell division (Mitchison, 2003). Such a control implies that cells monitor their size and feed this information into the cyclin-dependent kinases (CDKs) that drive the cell cycle. However, although a range of mechanisms have been proposed as to how cell size might be monitored and fed into the CDK mitotic cell cycle control, there is no agreement as to how the molecular mechanisms operate (Martin, 2009; Rhind, 2018; Schmoller and Skotheim, 2015).

One of the most fully investigated cell types in this regard is the fission yeast *Schizosaccharomyces pombe*. This single-celled eukaryote is rod shaped and grows by linear extension to 13–14 µm in length when the cell divides into two equally sized daughter cells (Mitchison and Nurse, 1985; Wood and Nurse, 2015). It has been shown that cell size at division is determined by the size at which cells activate the mitotic CDK, a complex between the Cdc2 protein kinase and the Cdc13 activating cyclin. Experiments monitoring the subsequent cell cycle after cells have divided at a range of sizes indicate that cells that are large at the start of the cell cycle proceed to mitosis more rapidly than cells that are small at the start of the cell cycle (Fantes, 1977). This demonstrates that there is a cell size homeostasis mechanism in place that corrects for deviations in cell size from the population mean, a correction that is made mostly within one cell cycle. Several size control mechanisms have been proposed to work in fission yeast. A cell pole-to-middle gradient formed by the DYRK kinase Pom1 has been proposed to relay size information to cell cycle regulators (Allard et al., 2019; Gerganova et al., 2019; Martin and Berthelot-Grosjean, 2009; Moseley et al., 2009). Alternatively, the correlation between a cell's surface and the number of membrane-associated Cdr2 nodes, a positive regulator of mitosis, has been argued to confer size-dependent cell division (Pan et al., 2014). Other models invoke that size homeostasis is driven by the accumulation of an unstable mitotic activator that is expressed in a size-dependent manner. The tyrosine phosphatase Cdc25, which dephosphorylates and activates CDK, has been proposed as such an unstable activator (Keifenheim et al., 2017). Similarly, the levels of mitotic cyclin B Cdc13 have been shown to scale with cell size and impact on the likelihood of cell division (Patterson et al., 2019).

To inform what molecular mechanisms are involved in this cell size homeostatic control, we have identified the genes involved using genome-wide genetic screens. A collection of fission yeast gene deletion mutants has been constructed (Kim et al., 2010), and ∼3000 mutants from this collection, covering 82% of the non-essential genes of fission yeast, have been screened to identify mutants that advance cells prematurely into mitosis at a smaller size and thus are rate limiting for the onset of mitosis (Navarro and Nurse, 2012). This screen identified 18 genes that when deleted advance cells into mitosis, and so are potential candidates for involvement in the cell size homeostatic mechanism. It should be noted that mutants that enter into mitosis with a larger size are not likely to be useful candidates, because a gene that is required for any aspect of cell cycle progression, if defective, can delay onset of mitosis. A second screen has been carried out in diploid heterozygous mutants of 565 genes required for successful completion of the cell cycle, identified from 4844 of the fission yeast genes (Hayles et al., 2013; Moris et al., 2016). This haploinsufficiency screen identified genes that, when expressed at a lower level, advance or delay mitotic onset. The fact that a relatively small change in gene product level, generally to ∼50% of wild-type, alters cell size at mitosis makes these genes also potential candidates for involvement in the cell size homeostatic mechanism. A total of 17 haploinsufficient genes were found (Moris et al., 2016), three of which overlap with the first screen, identifying 32 genes in total.

In this study we have undertaken a third screen to identify genes that when deleted increase the variability in the size of cells that undergo mitosis. Such a phenotype could indicate that the loss of the gene function leads to less accurate monitoring of cell size, potentially implicating that gene in the size-monitoring mechanism. This screen identified several candidates, the strongest of which were *mga2* and *cid12*. Deletion of *mga2* influences lipid composition and the nuclear membrane, doubles the coefficient of variation (CV) of cell size at mitosis and results in cells being unable to correct cell size deviations within one cell cycle.

## RESULTS

### Deletion mutants with increased cell size variability at division

**Fig. 1.**
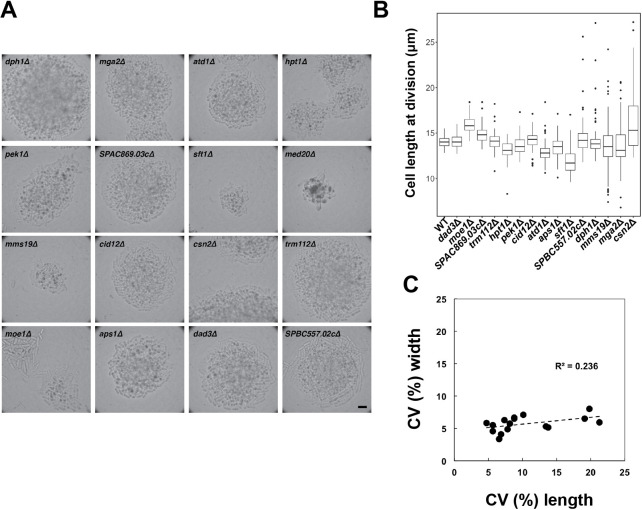
**Screen for gene deletions that cause increased variability in cell length at division.** (A) Micro-colonies formed by the mutant candidates analysed. Cells growing in YE4S liquid medium were streaked out to single colonies on YE4S plates. Micro-colonies were imaged after 16 h of incubation at 32°C. Scale bar: 20 μm. (B) Box-and-whisker plots showing cell length at division of the indicated mutants and the control strain (WT). Between 50 and 133 cells (exact sample sizes given in Table 1) from cultures growing in exponential phase in YE4S medium at 32°C were measured for each strain. Boxes are delimited by the first quartile, median and third quartile, while whiskers mark maximum and minimum values within the 10–90% range. Values outside this range are shown as individual dots. (C) Correlation between CV of cell length and CV of cell width at division for each of the mutant candidates analysed. Dashed line shows linear regression between the CV of cell width at division and the CV of cell length at division.

To identify fission yeast mutants with increased cell size variability at mitosis and division, we re-analysed data from a near genome-wide screen of cell size regulators (Navarro and Nurse, 2012). In this screen, overall cell growth and cell size at division were visually assessed from more than 3000 gene deletion mutants, covering 82% of all non-essential genes. Sixteen mutants originally annotated as ‘variable cell size’ were re-isolated from the haploid fission yeast deletion collection and examined at micro-colony stage of 50–100 cells on solid medium (YE4S medium, 32°C) (Fig. 1A). Fifteen mutants were able to form colonies under these conditions. One of these, the *med20* gene deletion strain, appeared sick (Fig. 1A) and contained multi-septated cells. For the remaining mutants, visual inspection confirmed the previous assessment of apparent higher cell size variability.

**Table 1.**
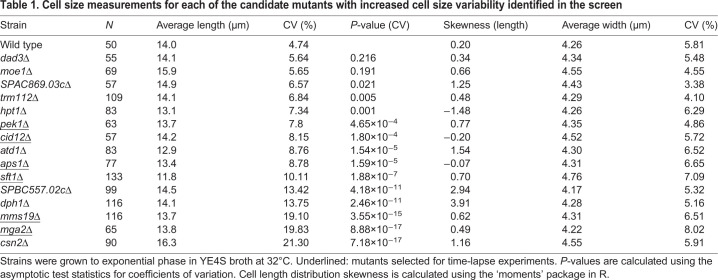
**Cell size measurements for each of the candidate mutants with increased cell size variability identified in the screen**

The higher cell size variability was not a consequence of primarily asymmetrical cell division, as all mutants divided giving rise to two roughly equally sized daughter cells. To further characterize their phenotype, these mutants were grown in liquid culture (YE4S medium, 32°C) and cell length at division during the exponential growth phase of the culture was measured in 50–133 cells (Fig. 1B, Table 1). The CV in size at division ranged from ∼6% to ∼21%, compared to 5% in the wild-type strain (Table 1). The increase in CV was accompanied in some cases by a change in the average cell length at division; four of the mutants had an average cell length at division at least one micrometre larger or smaller than that of the wild-type strain. The remaining candidates had an average cell length at division within one micrometre of that of the wild-type strain. The weak correlation between the CV values of cell length and cell width at division (Fig. 1C), suggested that variation in size mainly affected cell length and not cell width.

From this initial screen, we selected mutants with CVs of cell length at division that were significantly increased compared with that of wild type (*P*<0.001) and that also exhibited a symmetrical or near symmetrical cell length distribution (skewness −1 to +1; Table 1). The latter condition was applied to avoid selecting for mutants with increased CV due to partial activation of a cell cycle checkpoint, such as the DNA damage checkpoint, which would lead to cell-cycle-arrested elongated cells and thus an asymmetrical cell length distribution skewed towards larger cells. Six mutants, *pek1*Δ (also known as *skh1*Δ), *cid12*Δ, *aps1*Δ, *sft1*Δ, *mms19*Δ and *mga2*Δ, satisfied these criteria and were further characterized in time-lapse experiments.

### *mga2* and *cid12* deletions disrupt cell size homeostasis

**Fig. 2.**
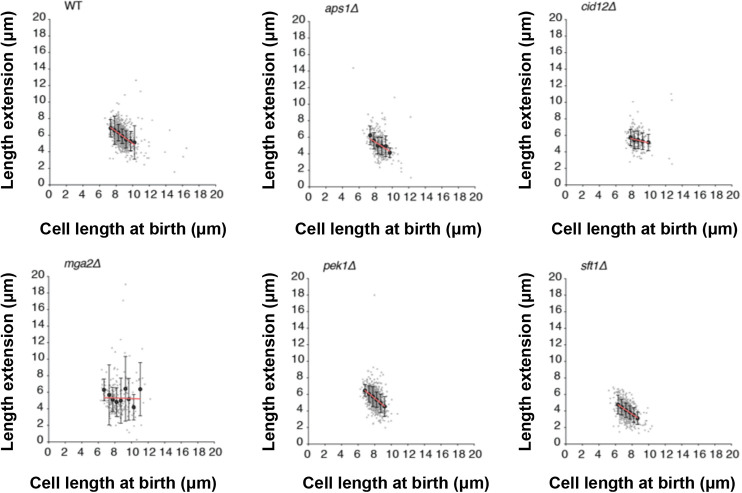
**Single-cell size homeostasis analysis of candidate mutants.** Cell length at birth plotted against length extension for the strains indicated in each plot. Measurements taken from bright-field time-lapse images of 223–895 cells for each strain (exact sample sizes given in Table 2). Length extension calculated from birth and division length measurements. Small light grey dots show individual cells. Larger dark grey points show mean values of cohorted data. Data are grouped into cohorts of 0.5 μm increments of cell length at birth. The linear regression analysis, as shown by the red line, is based on the raw non-cohorted data. Bars represent s.d. Cells were grown in YE4S medium at 32°C in a microfluidic chamber.

The strength of the cell size homeostatic control mechanism can be revealed by plotting the birth length (BL) of each individual cell of a population against how much the cell extends (E) during the cell cycle (BL/E plot; Fantes, 1977) (Fig. 2). For the wild-type strain, the points of this plot show a linear correlation with a slope value between −0.7 and −0.8, indicative that fission yeast cells can correct deviations in cell size mostly within one cell cycle. In contrast, mutants with a defective cell size control mechanism show less pronounced slopes (Fantes, 1977; Sveiczer et al., 1996). We applied this cell size homeostasis analysis to the six mutant strains selected from the initial screen in liquid medium. Cells were grown in microfluidic chips within a temperature-controlled microscope chamber and bright-field images were taken every 10 min over 12 h. In total, ∼200–900 cell cycles from each strain were analysed, and birth length, extension length during the cell cycle and cell cycle duration were extracted from the movies (Table 2, Fig. 2). We noticed that the CV of the wild-type strain grown under these conditions was higher than that of cells grown in liquid medium in a flask (8% versus 4.7%), presumably due to the effects of growth in a microfluidic chamber and/or phototoxicity during the imaging procedure.

**Table 2.**
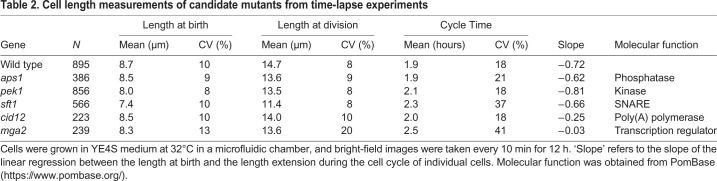
**Cell length measurements of candidate mutants from time-lapse experiments**

The slope values of the BL/E plots for three deletion mutants, *aps1*Δ, *pek1*Δ and *sft1*Δ, were similar to that of the wild type, and the CV for cell size at division of these mutants grown in the microfluidics chamber was not increased compared with that of the wild-type strain (Fig. 2, Table 2). Therefore, these deletion mutants were discarded for further investigation. The *mms19*Δ mutant was also discarded because the time-lapse experiment revealed that these mutant cells re-initiated growth before the septum was resolved (Fig. S1). This feature could explain the increased cell size variability observed.

**Fig. 3.**
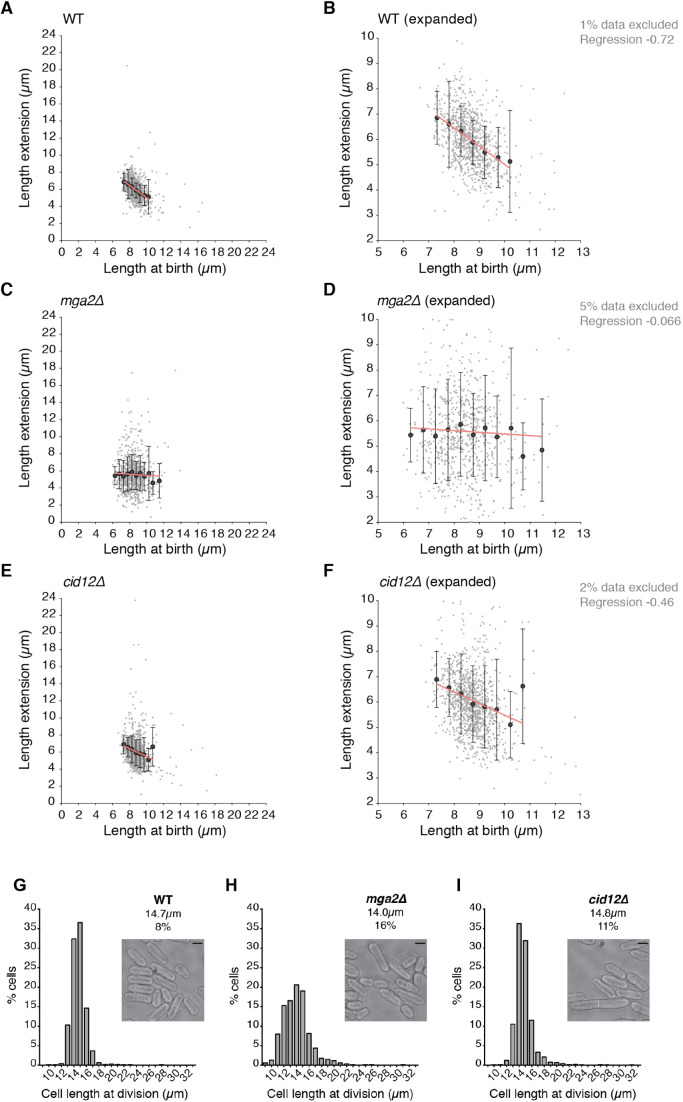
**Cells with *mga2* or *cid12* deletions show reduced cell size homeostasis.** (A,C,E) Cell length at birth plotted against length extension for the strains indicated. Measurements taken from bright-field time-lapse images of 895 (wild type, WT; A), 777 (*mga2*Δ; C) and 1138 (*cid12*Δ; E) cells. Length extension calculated from birth and division length measurements. Small light grey dots show individual cells. Larger dark grey points show mean values of cohorted data. Data are group into cohorts of 0.5 μm increments of cell length at birth. The linear regression analysis, as shown by the red line, is based on the raw non-cohorted data. Bars represent s.d. All cells were grown in YE4S medium at 32°C in a microfluidic chamber. (B,D,F) Plots as in A, C and E, respectively, with reduced axis scales for clarity. The percentage of data outside scale is indicated on the right along with the regression slope. (G–I) Frequency distributions of cell length at division for (G) WT, (H) *mga2*Δ and (I) *cid12*Δ (I), showing the same populations as plotted in A,C,E. Cells were grown in YE4S medium at 32°C in a microfluidic plate. Bin size: 1 µm. The mean cell length at division and the coefficient of variation are reported for each strain. Images show representative cells of each strain. Scale bars: 5 µm.

Two mutant strains, *cid12*Δ and *mga2*Δ, were retained. These showed higher slope values in the BL/E plots than the wild-type strain (−0.25 and −0.03, respectively) and larger variation in length extension within each cohort of birth lengths (Fig. 2). Cid12 is a poly(A) polymerase involved in RNA silencing and chromosome segregation (Motamedi et al., 2004; Win et al., 2006), whereas Mga2 is a transcription regulator involved in fatty acid metabolism (Burr et al., 2016, 2017). To rule out any effects of the auxotrophies present in the original *mga2*Δ and *cid12*Δ strains, new gene deletions were generated in the wild-type background *972 h^−^*. The cell length at division of the new mutant strains was measured in time-lapse experiments, extending the number of cycles analysed (777 cells for *mga2*Δ and 1138 cells for *cid12*Δ) (Fig. 3). Similar to the original strains, the prototrophic *mga2*Δ and *cid12*Δ mutants showed lower BL/E slope values (−0.066 and −0.46, respectively; Fig. 3A–F) and higher CV of lengths at division (16% and 11%, respectively; Fig. 3G–I) than the wild-type strain (BL/E slope of −0.72 and CV of 8%). These results confirm that both the *mga2*Δ and *cid12*Δ mutants have defects in the cell size homeostasis mechanism.

### Mga2 is involved in nuclear membrane homeostasis

**Fig. 4.**
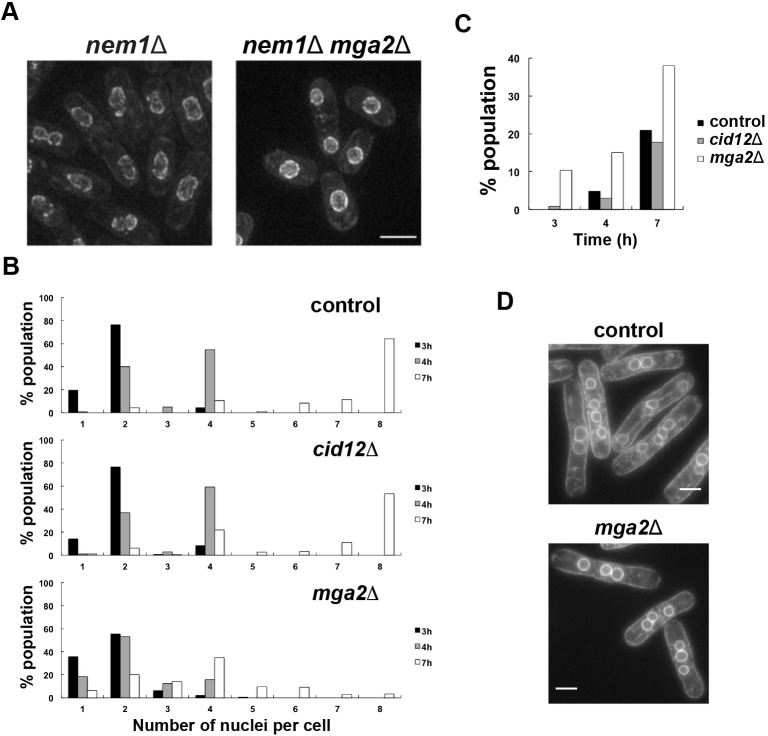
***mga2* deletion suppresses over-proliferated nuclear envelope in the *nem1* deletion mutant and leads to asynchronous nuclear divisions in the *cdc11-119* background.** (A) *mga2* deletion suppresses the nuclear mis-shape phenotype of the *nem1*Δ mutant. Nuclear envelope is imaged using Cut11–GFP marker. Scale bar: 5 µm. (B) Quantification of nuclei per cell, represented as a percentage of the population. Cells were incubated at 36.5°C for a total of 8 h, with nuclei per cell quantified after 3, 4 and 7 h of incubation. Number of nuclei per cell was determined from 894 cells (control strain, FN391), 627 cells (*mga2*Δ, LW314) and 768 cells (*cid12*Δ, LW313) across all time points. (C) Percentage of cells showing asynchronous nuclear divisions (three, five, six or seven nuclei) at 3, 4 and 7 h after the temperature rise. The ‘% population’ values shown were calculated using the total of cells with two or more nuclei (data from B). (D) Representative pictures of control and *mga2*Δ mutant cells showing different numbers of nuclei per cell. Nuclei were visualized using the endoplasmic reticulum marker Elo2–mCherry. Scale bars: 5 µm.

Mga2 is a transcriptional factor involved in triacylglycerol and glycerophospholipid synthesis, analogous to mammalian SREBP-1 (also known as SREBF1). Most Mga2 transcriptional targets are genes related to lipid metabolism (Burr et al., 2016), and the *mga2* mutation results in an increase in fatty acid saturation and aberrant membrane transport (Burr et al., 2017). We investigated whether *mga2* genetically interacted with *nem1.* Nem1 is the catalytic subunit of a phosphatase involved in the activation of lipin (Ned1), a phosphatide lipid phosphatase (Kume et al., 2017; Sajiki et al., 2018; Siniossoglou et al., 1998), and the *nem1* deletion leads to constitutive proliferation of nuclear membrane, resulting in cells with enlarged, floppy nuclear envelopes (Fig. 4A, left panel). Deletion of *mga2* in the *nem1* mutant largely reversed this floppy nuclear envelope and established a more normal spherical shape of the nucleus (Fig. 4A, right panel), suggesting that *mga2* is involved in nuclear membrane formation. It is possible that the *mga2* mutation reduces the available pool of cell membranes, compensating for the excess of nuclear membrane due to the *nem1* mutation.

### Asynchronous nuclear division in the mga2Δ mutant

To further investigate the role of *mga2* in the nuclear envelope and nuclear division, we analysed the impact of its deletion on the synchrony of nuclear divisions when cytokinesis was blocked. In such a situation, nuclei share a common cytoplasm and are subject to the same physiological conditions, and nuclei tend to divide synchronously. We used the *cdc11-119* mutation, which at restrictive temperature blocks cytokinesis while cells continue to grow and nuclei divide synchronously, resulting in cells with two, four or eight nuclei in each cell. Variability in the timing of the onset of mitosis in each nucleus, which may be the result of altered nuclear–cytoplasmic (N–C) transport of cell cycle factors, is manifested by the appearance of cells with a nuclei number outside this series. Cells of the *cdc11-119*, *cdc11-119 mga2*Δ and *cdc11-119 cid12*Δ strains were grown at restrictive temperature for 8 h, and images were taken after 3, 4, 7 and 8 h. Nuclei were visualised using the endoplasmic reticulum marker Elo2–mCherry, and the number of nuclei in each cell was determined across all time points for each strain. Fig. 4B shows this quantification. The most common numbers of nuclei per cell in the *cid12Δ* mutant were two, four and eight, similar to the control strain. Very few cells had three, five, six or seven nuclei. However, in the case of the *mga2*Δ mutant, although cells with two and four nuclei were predominant, cells carrying three, five or six nuclei were more abundant compared with their abundance in the control strain (Fig. 4B–D). This partial loss in nuclear division synchrony indicates that the phenotype of the *mga2* mutant is independent of cytokinesis, and is consistent with a role of the *mga2* gene in the N–C transport of cell cycle factors, which we discuss below.

### Interaction of *mga2* mutation with Cdk1 Tyr15 phosphorylation

Mitotic commitment is influenced in fission yeast by phosphorylation of the Tyr15 residue of Cdc2, the protein kinase component of CDK (Gould and Nurse, 1989; Morgan, 1997). Phosphorylation prevents mitosis and is regulated by action of the Wee1 kinase and Cdc25 phosphatase (Russell and Nurse, 1986, 1987), and to an extent by the Mik1 kinase (Lundgren et al., 1991). Reducing Tyr15 phosphorylation by mutation of *wee1* results in cells dividing at approximately half the size of the wild-type strain (Nurse, 1975). Mutation of the *mik1* gene does not result in any size phenotype, but is synthetically lethal with the *wee1* mutation. In addition, the *wee1* mutation increases the CV of size at division (Sveiczer et al., 1996) (Table 3). We therefore investigated the effect of the *mga2* mutation on CDK regulation.

**Table 3.**
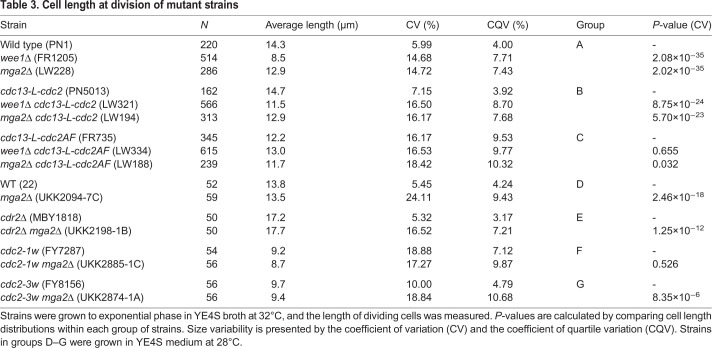
**Cell length at division of mutant strains**

**Fig. 5.**
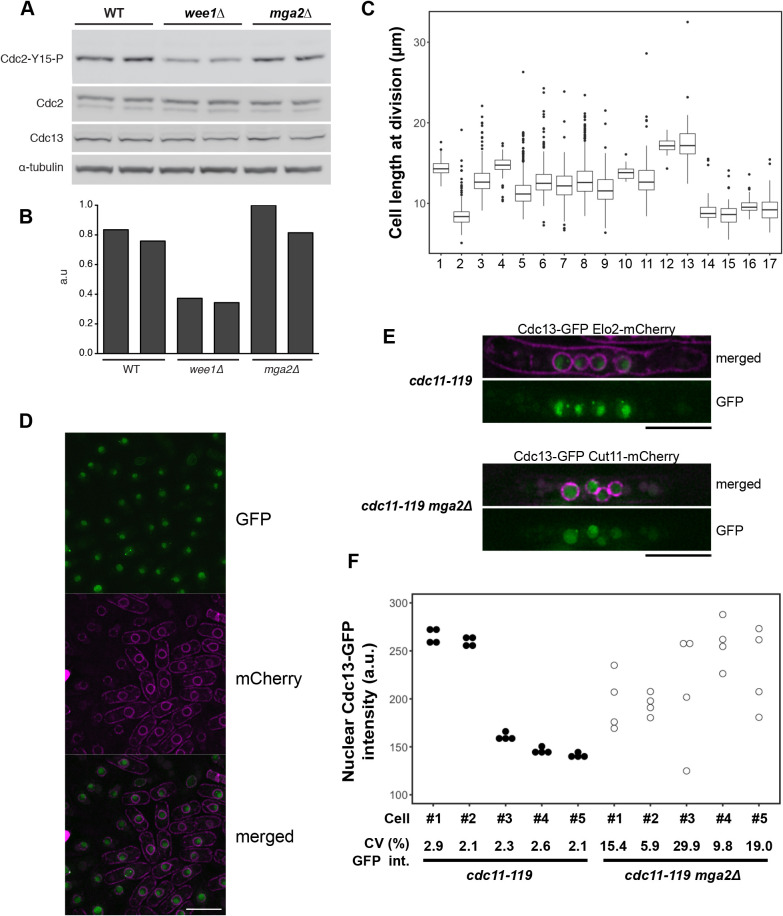
**Interaction of *mga2* mutation with Cdk1 Tyr15 phosphorylation and Cdc13 nuclear accumulation.** (A) Western blot for Tyr15-phosphorylated Cdc2 (Cdc2-Y15-P), Cdc2, Cdc13 and α-tubulin in the wild-type (WT), *wee1*Δ and *mga2*Δ strains. Each lane of the western blot represents a single experiment. (B) Quantification of phosphorylated-Tyr15 Cdc2 from the western blot shown in A, normalised against Cdc2 levels. (C) Box-and-whisker plots of cell length at division for the following strains: 1, WT; 2, *wee1*Δ; 3, *mga2*Δ; 4, *cdc13-L-cdc2*; 5, *wee1*Δ *cdc13-L-cdc2*; 6, *mga2*Δ *cdc13-L-cdc2*; 7, *cdc13-L-cdc2AF*; 8, *wee1*Δ *cdc13-L-cdc2AF*; 9, *mga2*Δ *cdc13-L-cdc2AF*; 10, WT; 11, *mga2*Δ; 12, *cdr2*Δ; 13, *cdr2*Δ *mga2*Δ; 14, *cdc2-1w*; 15, *cdc2-1w mga2*Δ; 16, *cdc2-3w*; 17, *cdc2-3w mga2*Δ. Cells were grown in YE4S medium at 32°C (1–9) or 28°C (10–17). Sample size for each strain shown in Table 3. Boxes are delimited by the first quartile, median and third quartile, while whiskers mark maximum and minimum values within the 10–90% range. Values outside this range are shown as individual dots. (D) Cdc13–GFP localization in the wild type and *mga2*Δ mutant cells. Cdc13–GFP Elo2–mCherry and *mga2*Δ Cdc13–GFP Cut11–mCherry strains were grown at 28°C in YES medium to mid-exponential phase. Cells from both strains were mixed on the slide before imaging. Cells of each genetic background can be easily distinguished because the nuclear envelope and the cell membrane of *mga2^+^* cells are labelled by the Elo2–mCherry marker, while only the nuclear envelope is labelled in *mga2*Δ Cdc13–GFP Cut11–mCherry cells. Scale bar: 10 µm. (E) Cdc13–GFP localization in the multinuclear system *cdc11-119*. *cdc11-119 cdc13–GFP elo2–mCherry* and *cdc11-119 mga2*Δ *cdc13–GFP cut11–mCherry* strains were grown to mid-exponential phase at 28°C in YES medium and then transferred to 36°C for 5 h. Both *mga2* backgrounds have labelled nuclear envelopes, while *mga2*^+^ cells have the cell membrane also labelled. Scale bar: 10 μm. (F) Quantification of nuclear Cdc13–GFP signal in five randomly selected four-nuclei cells for each genetic background. The GFP intensity inside each nucleus for four-nuclei cells is shown. The coefficient of variation in nuclear GFP intensities per cell is shown below.

To investigate this, we determined Tyr15 phosphorylation levels in the *mga2*Δ mutant using western blotting with specific antibodies against the phosphorylated Tyr15 residue (Fig. 5A,B). The *mga2*Δ mutant showed similar levels of Tyr15 phosphorylation as the wild-type strain, whereas these were reduced in the *wee1* mutant, as expected. The remaining Tyr15 phosphorylation detected in the *wee1* mutant is probably caused by the Mik1 kinase (Lundgren et al., 1991). The cyclin Cdc13 levels remained similar in all three strains. These results suggest that the increased CV of cell size at division of the *mga2*Δ mutant is not due to a significant defect in the Tyr15 phosphorylation regulation of CDK. To further investigate the interaction between Mga2 and CDK Tyr15 phosphorylation, the effect of the *mga2*Δ mutation was analysed in a background carrying a Cdc2 devoid of Tyr15 phosphorylation, as a result of mutation of the Tyr15 residue to Phe15. Additionally, Thr14 was mutated to Ala14, because this residue also becomes phosphorylated, albeit to a lower level (Den Haese et al., 1995). This *cdc2AF* mutation produces severe growth defects that are mostly suppressed when the Cdc2AF protein is fused to its mitotic Cdc13 cyclin partner and expressed using the *cdc13* promoter (Coudreuse and Nurse, 2010), so a strain whose cell cycle is driven by this fusion protein was used for these experiments. As a control, we also crossed the *wee1*Δ mutation into this background (Fig. 5C, Table 3). We noticed that some of the small size mutants used in this study tended to generate larger cells spontaneously, most likely diploid cells, which distorted the CV, because this coefficient is sensitive to outliers. Therefore, the coefficient of quartile variation (CQV) is also shown in Table 3 as a measure of relative data dispersion that is not sensitive to outliers. The *wee1*Δ and *mga2*Δ mutations increased the CV of cell size at division by greater than 2-fold (and increased the CQV greater than 1.8-fold) in both the wild-type strain and a *cdc13-L-cdc2* background carrying a fusion Cdc13–Cdc2 protein in which Tyr15 phosphorylation can take place. The non-phosphorylatable *cdc13-L-cdc2AF* strain had a CV of cell size at division of 16.2%, which was similar to that of the *wee1*Δ *cdc13-L-cdc2* and *mga2*Δ *cdc13-L-cdc2* strains. Deletion of *wee1* and of *mga2* in the fusion *cdc13-L-cdc2AF* background increased the CV by only 0.3% and 2.2%, respectively, and the CVs of the three strains were not statistically different (using a threshold of *P*<0.01) (Table 3). Smaller marginal increases in cell size variability were seen using the CQV index. In summary, these results show that absence of Tyr15 phosphorylation regulation by a *wee1* mutation or by expression of a mutant non-phosphorylatable CDK results in a high CV of cell size at division, similar to that shown by the *mga2* mutation. Furthermore, the *mga2* mutation does not significantly increase the CV of cell size at division of the non-phosphorylatable CDK strain. However, the large cell-to-cell size variability observed in the non-phosphorylatable CDK strain might obscure some effect of the *mga2* mutation in this background. To provide further insight into the role of CDK Tyr15 phosphorylation in the *mga2* mutant phenotype, we introduced the *mga2* mutation into the *cdc2-1w* and *cdc2-3w* mutant backgrounds. The *cdc2-1w* (G146D) and *cdc2-3w* (C67Y) strains divide at a smaller cell size than the wild type and with higher variability (Sveiczer et al., 1996). CDK Tyr15 phosphorylation still occurs in these mutants (Gould et al., 1990), but they respond differently to the Tyr15 phosphorylation regulators Wee1 and Cdc25 (Russell and Nurse, 1987). The *cdc2-3w* background suppresses the loss of the mitotic activator Cdc25, and is unable to arrest when DNA replication is blocked (Enoch and Nurse, 1990). In contrast, the *cdc2-1w* background, which is largely insensitive to the mitotic inhibitor Wee1, still arrests upon DNA replication blockage. Compared to the T14A Y15F CDK (Cdc13-L-Cdc2AF) strain, which is not regulated by Tyr 15 phosphorylation, these mutants grew more healthily and showed a narrower dispersion of cell sizes at division. The size variability at division of both *cdc2* mutants, but particularly *cdc2-3w*, as measured by the CQV, increased when the *mga2* gene was deleted (Table 3, Fig. 5C). The increase in variability of cell size at division in both mutant backgrounds, where Tyr15 phosphorylation regulation is altered in different ways, would suggest that the *mga2* product is not involved in regulation of CDK by Tyr15 phosphorylation, or at least not directly, but rather it affects other additional regulatory mechanisms.

Also implicated in mitotic CDK control is the Pom1 DYRK protein kinase/SAD-family protein kinase Cdr2 pathway, which inhibits the Wee1 protein kinase that phosphorylates Tyr15 of Cdc2 (Facchetti et al., 2019; Martin and Berthelot-Grosjean, 2009; Moseley et al., 2009). We therefore also tested the effects of the *mga2* deletion on cell size variability at division of cells lacking *cdr2.* The *mga2* deletion increased the CV of cell size at division of the *cdr2* deletion cells ∼3-fold, similar to what was seen in *cdr2^+^* cells (Table 3, Fig. 5C). Therefore, cells lacking Mga2 still increase variability in cell size at mitosis when Cdr2 is absent.

### Uncoordinated nuclear accumulation of CDK in the *mga2* mutant

During the cell cycle, CDK gradually accumulates in the nucleus and spindle pole body (SPB) (Decottignies et al., 2001). In prophase and metaphase cells, the CDK also decorates the growing spindle until sister chromatid separation, after which point CDK nuclear signal is reduced. Cdc2 requires at least one B-type cyclin (Cig1, Cig2 or Cdc13) to accumulate in the nucleus after G1/S, with Cdc13 being essential for nuclear, SPB and spindle CDK accumulation during G2 and early phases of mitosis. We investigated the effect of *mga2* mutation on the cellular localization of Cdc13, which ultimately controls the localization of CDK. We used a strain in which the *cdc13* gene is fused to GFP and that, in addition, expresses either a Cut11–mCherry fusion, which labels the nuclear envelope, or an Elo2–mCherry fusion, which labels the nuclear membrane and cell membrane. The *mga2* gene was deleted in the *cdc13–GFP cut11–mCherry* background, and Cdc13–GFP cellular localization was compared to *mga2^+^ cdc13–GFP Elo2–mCherry* cells. In both *mga2^+^* and *mga2* mutant cells, Cdc13–GFP signal increased in the nucleus as cells increased in size, and a bright dot on the nucleus surface, most likely corresponding to the SPB, could be distinguished (Fig. 5D). Short spindles were also decorated with Cdc13–GFP. No differences in cyclin localization were observed between *mga2^+^* and *mga2* mutant cells. Cdc13–GFP cellular localization was also studied in the multinuclear system *cdc11-119*. The *mga2* mutation was introduced into a *cdc11-119 cdc13–GFP* background, and Cdc13 localization and accumulation were studied in four-nuclei cells. The Cdc13–GFP cellular distribution in the multinuclear system was similar to cells with a single nucleus, regardless the *mga2* background. However, the Cdc13–GFP levels across the four nuclei within the same cell followed different patterns (Fig. 5E,F). In the *mga2*^+^ background, Cdc13–GFP accumulated in each of the four nuclei within the same cell to very similar levels. In contrast, the Cdc13–GFP signal in the *mga2* mutant showed high variability across the four nuclei of the same cell, in all the cells analysed. The high variability in Cdc13–GFP nuclear accumulation, together with the partial asynchronous nuclear division seen in the multinuclear *mga2* mutant, supports a role for Mga2 in nuclear transport or stability of the mitotic CDK.

## DISCUSSION

The size uniformity of dividing fission yeast cells has been much studied, but the mechanism of cell size control is still not understood. The search for mutants with variable cell size, such as the ones described here, could help to distinguish between truly cell-size-sensing mechanisms and cell cycle regulators. Mutations in the latter factors frequently result in cells that divide at smaller or larger sizes, but intriguingly these still divide with a uniform cell size. In this paper, we have identified two genes that when deleted increase variability in cell size at onset of mitosis: *mga2*, which encodes a transcriptional factor involved in triacylglycerol and glycerophospholipid synthesis (Burr et al., 2016, 2017), and *cid12*, which is a poly(A) polymerase involved in RNA silencing and chromosome segregation (Motamedi et al., 2004; Win et al., 2006). Both of these gene deletions are also altered in the speed with which cells correct deviations in cell size at the onset of mitosis. Unlike wild-type cells, deviations are not corrected within one cell cycle, and in the case of *mga2*Δ cells correction is particularly slow (Figs 2,3). The *mga2*Δ cells essentially add on a constant size amount each cell cycle, which will return cells to the population mean but will take several cell cycles to do so. The shift from a ‘sizer’ to an ‘adder’ mechanism as a consequence of the deletion of a single gene – *mga2* – has implications for thinking about appropriate molecular mechanisms.

The rather general nature of the defects induced by alterations in poly(A) polymerization (*cid12*) and regulation of lipid synthesis (*mga2*) make it difficult to be specific about the molecular mechanisms involved in the monitoring of cell size and CDK activation at the onset of mitosis. However, some comments can be made about Mga2, given that lipid synthesis will influence membrane formation and that the nuclear membrane plays a critical role in both onset and progression through mitosis (Makarova et al., 2016; Saitoh et al., 1996; Takemoto et al., 2016). In fission yeast, mitosis is closed, and so alterations in membrane composition could influence properties such as membrane fluidity and the ability of the nucleus to rapidly extend during mitosis. In addition, the nuclear membrane plays an important role in chromosome positioning. For example, telomeres are tethered to the nuclear envelope by the interaction of the shelterin complex with the inner nuclear envelope protein Bqt4 (Chikashige et al., 2009), and subtelomere regions by the association between the Fft3 chromatin remodeller and the nuclear membrane protein Man1 (Steglich et al., 2015). At this point it is not known whether chromosome nuclear position or chromosome dynamics are altered in the *mga2* mutant, nor whether chromosome positioning has an impact on the accuracy of mitotic onset. However, more important, and generalizable to other eukaryotic cells with an open mitosis, is the implication of N–C transport in the control of the onset of mitosis (Chua et al., 2002; Moore et al., 1999; Strauss et al., 2018). Seven genes involved in N–C transport in fission yeast change cell size at mitotic onset when deleted (Moris et al., 2016), and it is possible that the Mga2 transcription factor affects the nuclear membrane in a manner that disturbs N–C transport and that this introduces variability into the transport of CDK or its regulators into the nucleus, increasing variability of cell size at mitosis and cell size homeostasis. In fact, we observed partial loss of nuclear division synchrony in the absence of Mga2 in a multinuclear model (Fig. 4), which we argue could be the result of a defective N–C transport of cell cycle factors. In this same multinuclear system, we also observed high variability in Cdc13–GFP nuclear levels when the *mga2* gene was deleted (Fig. 5F), which supports the hypothesis that the Mga2 transcription factor may affect CDK N–C transport. To probe this further, we examined the CV of cell size at division in heterozygote deletion diploid strains of fission yeast altered in N–C transport, which are listed in the Supplemental material of Moris et al. (2016). Of six nucleoporin genes involved in N–C transport, four of the heterozygous deletion diploids showed a significantly increased CV of cell size at division compared with that of the wild type (CV of 11.1%). These were *nup97* (CV of 17.5%), *nup18*6 (CV of 16.0%), *nsp1* (CV of 13.8%) and *nup184* (CV of 13.3%), all of which encode proteins in the nuclear pore. This provides support for the speculation that N–C transport of CDK regulators may influence the accuracy of cell size control at the onset of mitosis.

The discovery of two genes, *mga2* and *cid12*, that introduce increased variability into the cell size homeostatic control, brings the total number of genes potentially involved in this mechanism in fission yeast defined so far to 34. These genes are candidates for being required for the proper monitoring of cell size and transmitting this information to the activation of the mitotic CDK control mechanism, resulting in a larger variation of cell size at mitosis and cell division, possibly operating through transport of CDK into the nucleus.

## MATERIALS AND METHODS

### Yeast strains and growth conditions

Strains are listed in Table S1. Standard *S. pombe* media and methods were used (Moreno et al., 1991). The viable set of a near genome-wide *S. pombe* haploid gene deletion collection was used for the initial screen (Kim et al., 2010; Navarro and Nurse, 2012). Further strains were constructed using PCR and homologous recombination (Bähler et al., 1998). All cells were grown at 32°C in YE4S, unless otherwise stated. The *mga2*Δ strain was somewhat variable in terms of its growth in liquid culture, especially coming out of stationary phase. Growth rate was reduced when cells were diluted too much, and was improved when the culture was unshaken and incubated at 28°C. For the generation of multinucleate cells, strains with the temperature-sensitive *cdc11-119* allele were used. Incubation at the restrictive temperature, 36.5°C, results in cells undergoing rounds of nuclear division without cytokinesis.

### Imaging and cell length analysis

Primary screening of microcolonies growing on YE4S agar was carried out using a Zeiss Axioskop 40 microscope equipped with a 20×/0.4 NA objective plus an additional 1.8× magnification. A Sony NEX-5N camera was used for image acquisition. For secondary screening, length measurements of candidates in liquid culture were carried out. Cells were either measured in liquid culture on a glass slide or transferred to a Y04C microfluidic plate (CellASIC). Images were acquired using the DeltaVision Elite (described below) or a Zeiss Axioskop 40 microscope equipped with a 63×/1.4 NA objective and a Zeiss AxioCam MRm camera. Cell length was either measured from bright-field images or images of live cells stained with Calcofluor (Sigma). Measurements were taken using the PointPicker plugin of ImageJ (National Institutes of Health), and septated cells were chosen as cells with a visible septum. Length in pixel number was converted to micrometres.

Fluorescence imaging was carried out using a DeltaVision Elite microscope (Applied Precision) comprised of an Olympus IX71 wide-field inverted fluorescence microscope, an Olympus Plan APO 60×1.42 NA oil objective and a Photometrics CoolSNAP HQ2 camera (Roper Scientific) in an IMSOL ‘ImCubator’ Environment Control System set at 32°C. Images were acquired in 0.2 or 0.4 μm *z*-sections over 4.8–5.0 μm using SoftWoRx (Applied Precision) and processed using SoftWoRx and Huygens. Images are maximum intensity projections of deconvolved images. For quantitative Cdc13 localization, cells expressing Cdc13–GFP were imaged in 11 *z*-sections of 0.3 µm each, and the merged images were analysed using ImageJ. The fluorescence intensity of nuclear Cdc13–GFP is the average of seven measurements of a square area contained within the nucleus.

### Single-cell studies

The ONIX microfluidic perfusion system from CellASIC was used for single-cell analysis. Microfluidic plates were set up as described here: http://www.cellasic.com/ONIX_yeast.html. 50 μl of cell culture was loaded at a density of 1.26×10^6^ cells/ml, and cells were imaged in the 3.5 μm and 4.5 μm chambers. YE4S medium was used, and a flow rate of 3 psi (∼20 kPa) was maintained throughout the experiment. Time-lapse imaging was started 1 h after loading the plate and was carried out at 32°C using the DeltaVision Elite system described above. Images were acquired at 10 min intervals for 12 h. Cells were only measured during the first three generations after loading into the plate. Birth length was measured using the first frame in which two separate daughter cells were seen, and single cells were tracked through the frames until a septum formed, and this was taken as cell length at division. Cycle time was given as the time interval between these birth and division length measurements.

### Statistics

R (https://www.R-project.org/) was used for data analysis, data display and summary statistics. The coefficient of variation (CV=100×standard deviation/mean) and the coefficient of quartile variation [CQV=100× (Q3−Q1)/(Q3+Q1)] were used as relative measures of dispersion. A minimum sample size of 50 cells was established as the minimum number of observations that results in a 95% confidence interval no larger than ±50% of the wild-type standard deviation. The R package cvequality (version 0.1.3; https://cran.r-project.org/web/packages/cvequality/index.html) was used to test for significant differences between coefficients of variation of cell length, using the asymptotic test developed by Feltz and Miller (1996).

### Protein extracts and western blots

The levels of Cdc2 Tyr15 phosphorylation and Cdc13 protein were assessed using SDS-PAGE (NuPAGE Bis-Tris precast gels) and western blotting, according to the manufacturer's instructions (Life Technologies). Cells were quenched with 10% trichloroacetic acid and kept on ice for 30 min. Cells were pelleted by centrifugation and washed with acetone. Cell pellets were washed and resuspended in 100 µl lysis buffer [8 M urea, 50 mM ammonium bicarbonate, cOmplete Mini EDTA-free protease inhibitor cocktail (Roche), PhosSTOP phosphatase inhibitor cocktail (Roche)]. Cells were then disrupted in a FastPrep cell disrupter (FastPrep120) with acid-washed glass beads (0.4 mm, Sigma). The cell debris was pelleted and the supernatant was recovered as a protein extract. The antibodies listed below were used for the detection of proteins by western blotting. Signal was detected using SuperSignal West Femto Maximum Sensitivity Substrate (34095, Life Technologies) and imaged using an ImageQuant LAS 4000. Quantification was performed using ImageQuant TL software (GE Healthcare). Primary antibodies were used at the following concentrations: anti-Cdc13, 1:6000 (SP4, rabbit polyclonal; Moreno et al., 1989); anti-Cdc2, 1:250 (anti-PSTAIRE, rabbit polyclonal; Santa Cruz Biotech, SC-53); anti-Cdc2-Y15P, 1:500 [phospho-Cdc2 (Y15), rabbit polyclonal; #9111, Cell Signaling Technology]; anti-alpha tubulin (TAT1), 1:10,000 (mouse monoclonal; Woods et al., 1989). Secondary antibodies were used at the following concentrations: 1:25,000 horseradish peroxidase-conjugated donkey anti-rabbit (NA934V, GE Healthcare); 1:25,000 horseradish peroxidase-conjugated goat anti-mouse (STAR120P, AbD SeroTEC).

## Supplementary Material

Supplementary information

## References

[JCS251769C1] Allard, C. A. H., Opalko, H. E. and Moseley, J. B. (2019). Stable Pom1 clusters form a glucose-modulated concentration gradient that regulates mitotic entry. *eLife* 8, e46003 10.7554/eLife.46003 31050341 PMC6524964

[JCS251769C2] Bähler, J., Wu, J.-Q., Longtine, M. S., Shah, N. G., McKenzie, A., III, Steever, A. B., Wach, A., Philippsen, P. and Pringle, J. R. (1998). Heterologous modules for efficient and versatile PCR-based gene targeting in *Schizosaccharomyces* pombe. *Yeast* 14, 943-951. 10.1002/(SICI)1097-0061(199807)14:10<943::AID-YEA292>3.0.CO;2-Y 9717240

[JCS251769C3] Burr, R., Stewart, E. V., Shao, W., Zhao, S., Hannibal-Bach, H. K., Ejsing, C. S. and Espenshade, P. J. (2016). Mga2 transcription factor regulates an oxygen-responsive lipid homeostasis pathway in fission yeast. *J. Biol. Chem.* 291, 12171-12183. 10.1074/jbc.M116.723650 27053105 PMC4933267

[JCS251769C4] Burr, R., Stewart, E. V. and Espenshade, P. J. (2017). Coordinate regulation of yeast sterol regulatory element-binding protein (SREBP) and Mga2 transcription factors. *J. Biol. Chem.* 292, 5311-5324. 10.1074/jbc.M117.778209 28202541 PMC5392677

[JCS251769C5] Chikashige, Y., Yamane, M., Okamasa, K., Tsutsumi, C., Kojidani, T., Sato, M., Haraguchi, T. and Hiraoka, Y. (2009). Membrane proteins Bqt3 and −4 anchor telomeres to the nuclear envelope to ensure chromosomal bouquet formation. *J. Cell Biol.* 187, 413-427. 10.1083/jcb.200902122 19948484 PMC2779253

[JCS251769C6] Chua, G., Lingner, C., Frazer, C. and Young, P. G. (2002). The sal3+ gene encodes an importin-β implicated in the nuclear import of Cdc25 in Schizosaccharomyces pombe. *Genetics* 162, 689-703. 12399381 10.1093/genetics/162.2.689 PMC1462273

[JCS251769C7] Coudreuse, D. and Nurse, P. (2010). Driving the cell cycle with a minimal CDK control network. *Nature* 468, 1074-1079. 10.1038/nature09543 21179163

[JCS251769C8] Decottignies, A., Zarzov, P. and Nurse, P. (2001). In vivo localisation of fission yeast cyclin-dependent kinase cdc2p and cyclin B cdc13p during mitosis and meiosis. *J. Cell Sci.* 114, 2627-2640. 11683390 10.1242/jcs.114.14.2627

[JCS251769C9] Den Haese, G. J., Walworth, N., Carr, A. M. and Gould, K. L. (1995). The Wee1 protein kinase regulates T14 phosphorylation of fission yeast Cdc2. *Mol. Biol. Cell* 6, 357-484. 10.1091/mbc.6.4.371 7626804 PMC301198

[JCS251769C10] Enoch, T. and Nurse, P. (1990). Mutation of fission yeast cell cycle control genes abolishes dependence of mitosis on DNA replication. *Cell* 60, 665-673. 10.1016/0092-8674(90)90669-6 2406029

[JCS251769C11] Facchetti, G., Knapp, B., Flor-Parra, I., Chang, F. and Howard, M. (2019). Reprogramming Cdr2-dependent geometry-based cell size control in fission yeast. *Curr. Biol.* 29, 350-358.e4. 10.1016/j.cub.2018.12.017 30639107 PMC6345630

[JCS251769C12] Fantes, P. A. (1977). Control of cell size and cycle time in Schizosaccharomyces pombe. *J. Cell Sci.* 24, 51-67. 893551 10.1242/jcs.24.1.51

[JCS251769C13] Feltz, C. J. and Miller, G. E. (1996). An asymptotic test for the equality of coefficients of variation from k populations. *Stat. Med.* 15, 647-658. 10.1002/(SICI)1097-0258(19960330)15:6<647::AID-SIM184>3.0.CO;2-P 8731006

[JCS251769C14] Gerganova, V., Floderer, C., Archetti, A., Michon, L., Carlini, L., Reichler, T., Manley, S. and Martin, S. G. (2019). Multi-phosphorylation reaction and clustering tune Pom1 gradient mid-cell levels according to cell size. *eLife* 8, e45983 10.7554/eLife.45983 31050340 PMC6555594

[JCS251769C15] Gould, K. L. and Nurse, P. (1989). Tyrosine phosphorylation of the fission yeast cdc2^+^ protein kinase regulates entry into mitosis. *Nature* 342, 39-45. 10.1038/342039a0 2682257

[JCS251769C16] Gould, K. L., Moreno, S., Tonks, N. K. and Nurse, P. (1990). Complementation of the mitotic activator, p8°cdc25, by a human protein-tyrosine phosphatase. *Science* 250, 1573-1576. 10.1126/science.1703321 1703321

[JCS251769C17] Hayles, J., Wood, V., Jeffery, L., Hoe, K.- L., Kim, D.-U., Park, H.-O., Salas-Pino, S., Heichinger, C. and Nurse, P. (2013). A genome-wide resource of cell cycle and cell shape genes of fission yeast. *Open Biol.* 3, 130053 10.1098/rsob.130053 23697806 PMC3866870

[JCS251769C18] Keifenheim, D., Sun, X.-M., D'Souza, E., Ohira, M. J., Magner, M., Mayhew, M. B., Marguerat, S. and Rhind, N. (2017). Size-dependent expression of the mitotic activator Cdc25 suggests a mechanism of size control in fission yeast. *Curr. Biol.* 27, 1491-1497.e4. 10.1016/j.cub.2017.04.016 28479325 PMC5479637

[JCS251769C19] Kim, D.-U., Hayles, J., Kim, D., Wood, V., Park, H.-O., Won, M., Yoo, H.-S., Duhig, T., Nam, M., Palmer, G. et al. (2010). Analysis of a genome-wide set of gene deletions in the fission yeast Schizosaccharomyces pombe. *Nat. Biotechnol.* 28, 617-623. 10.1038/nbt.1628 20473289 PMC3962850

[JCS251769C20] Kume, K., Cantwell, H., Neumann, F. R., Jones, A. W., Snijders, A. P. and Nurse, P. (2017). A systematic genomic screen implicates nucleocytoplasmic transport and membrane growth in nuclear size control. *PLoS Genet.* 13, e1006767 10.1371/journal.pgen.1006767 28545058 PMC5436639

[JCS251769C21] Lundgren, K., Walworth, N., Booher, R., Dembski, M., Kirschner, M. and Beach, D. (1991). Mik1 and Wee1 cooperate in the inhibitory Tyrosine Phosphorylation of Cdc2. *Cell* 64, 1111-1122. 10.1016/0092-8674(91)90266-2 1706223

[JCS251769C22] Makarova, M., Gu, Y., Chen, J.-S., Beckley, J. R., Gould, K. L. and Oliferenko, S. (2016). Temporal regulation of Lipin activity diverged to account for differences in mitotic programs. *Curr. Biol.* 26, 237-243. 10.1016/j.cub.2015.11.061 26774782 PMC4728079

[JCS251769C23] Martin, S. G. (2009). Geometric control of the cell cycle. *Cell Cycle* 8, 3643-3647. 10.4161/cc.8.22.9891 19844171

[JCS251769C24] Martin, S. G. and Berthelot-Grosjean, M. (2009). Polar gradients of the DYRK-family kinase Pom1 couple cell length with the cell cycle. *Nature* 459, 852-856. 10.1038/nature08054 19474792

[JCS251769C25] Mitchison, J. M. (2003). Growth during the cell cycle. *Int. Rev. Cytol.* 226, 165-258. 10.1016/S0074-7696(03)01004-0 12921238

[JCS251769C26] Mitchison, J. M. and Nurse, P. (1985). Growth in cell length in the fission yeast Schizosaccharomyces pombe. *J. Cell Sci.* 75, 357-376. 4044680 10.1242/jcs.75.1.357

[JCS251769C27] Moore, J. D., Yang, J., Truant, R. and Kornbluth, S. (1999). Nuclear import of Cdk/cyclin complexes: identification of distinct mechanisms for import of Cdk2/cyclin E and Cdc2/cyclin B1. *J. Cell Biol.* 144, 213-224. 10.1083/jcb.144.2.213 9922449 PMC2132890

[JCS251769C28] Moreno, S., Hayles, J. and Nurse, P. (1989). Regulation of p34cdc2 protein kinase during mitosis. *Cell* 58, 361-372. 10.1016/0092-8674(89)90850-7 2665944

[JCS251769C29] Moreno, S., Klar, A. and Nurse, P. (1991). Molecular genetic analysis of fission yeast Schizosaccharomyces pombe. *Methods Enzym.* 194, 795-823. 10.1016/0076-6879(91)94059-L 2005825

[JCS251769C30] Morgan, D. O. (1997). CYCLIN-DEPENDENT KINASES: Engines, Clocks, and Microprocessors. *Annu. Rev. Cell Dev. Biol.* 13, 261-291. 10.1146/annurev.cellbio.13.1.261 9442875

[JCS251769C31] Moris, N., Shrivastava, J., Jeffery, L., Li, J.-J., Hayles, J. and Nurse, P. (2016). A genome–wide screen to identify genes controlling the rate of entry into mitosis in fission yeast. *Cell Cycle* 15, 3121-3130. 10.1080/15384101.2016.1242535 27736299 PMC5134717

[JCS251769C32] Moseley, J. B., Mayeux, A., Paoletti, A. and Nurse, P. (2009). A spatial gradient coordinates cell size and mitotic entry in fission yeast. *Nature* 459, 857-860. 10.1038/nature08074 19474789

[JCS251769C33] Motamedi, M. R., Verdel, A., Colmenares, S. U., Gerber, S. A., Gygi, S. P. and Moazed, D. (2004). Two RNAi complexes, RITS and RDRC, physically interact and localize to noncoding centromeric RNAs. *Cell* 119, 789-802. 10.1016/j.cell.2004.11.034 15607976

[JCS251769C34] Navarro, F. J. and Nurse, P. (2012). A systematic screen reveals new elements acting at the G2/M cell cycle control. *Genome Biol.* 13, R36 10.1186/gb-2012-13-5-r36 22624651 PMC3446289

[JCS251769C35] Nurse, P. (1975). Genetic control of cell size at cell division in yeast. *Nature* 256, 547-551. 10.1038/256547a0 1165770

[JCS251769C36] Pan, K. Z., Saunders, T. E., Flor-Parra, I., Howard, M. and Chang, F. (2014). Cortical regulation of cell size by a sizer cdr2p. *eLife* 3, e02040 10.7554/eLife.02040 24642412 PMC3956294

[JCS251769C37] Patterson, J. O., Rees, P. and Nurse, P. (2019). Noisy cell-size-correlated expression of cyclin B drives probabilistic cell-size homeostasis in fission yeast. *Curr. Biol.* 29, 1379-1386.e4. 10.1016/j.cub.2019.03.011 30955932 PMC6488275

[JCS251769C38] Rhind, N. (2018). Cell size control via an unstable accumulating activator and the phenomenon of excess mitotic delay. *BioEssays* 40, 1-5. 10.1002/bies.201700184 29283187

[JCS251769C39] Russell, P. and Nurse, P. (1986). cdc25+ functions as an inducer in the mitotic control of fission yeast. *Cell* 45, 145-153. 10.1016/0092-8674(86)90546-5 3955656

[JCS251769C40] Russell, P. and Nurse, P. (1987). Negative regulation of mitosis by wee1+, a gene encoding a protein kinase homolog. *Cell* 49, 559-567. 10.1016/0092-8674(87)90458-2 3032459

[JCS251769C41] Saitoh, S., Takahashi, K., Nabeshima, K., Yamashita, Y., Nakaseko, Y., Hirata, A. and Yanagida, M. (1996). Aberrant mitosis in fission yeast mutants defective in fatty acid synthetase and acetyl CoA carboxylase. *J. Cell Biol.* 134, 949-961. 10.1083/jcb.134.4.949 8769419 PMC2120970

[JCS251769C42] Sajiki, K., Tahara, Y., Uehara, L., Sasaki, T., Pluskal, T. and Yanagida, M. (2018). Genetic regulation of mitotic competence in G_0_ quiescent cells. *Sci. Adv.* 4, eaat5685 10.1126/sciadv.aat5685 30116786 PMC6093628

[JCS251769C43] Schmoller, K. M. and Skotheim, J. M. (2015). The biosynthetic basis of cell size control. *Trends Cell Biol.* 25, 793-802. 10.1016/j.tcb.2015.10.006 26573465 PMC6773270

[JCS251769C44] Siniossoglou, S., Santos-Rosa, H., Rappsilber, J., Mann, M. and Hurt, E. (1998). A novel complex of membrane proteins required for formation of a spherical nucleus. *EMBO J.* 17, 6449-6464. 10.1093/emboj/17.22.6449 9822591 PMC1170993

[JCS251769C45] Steglich, B., Strålfors, A., Khorosjutina, O., Persson, J., Smialowska, A., Javerzat, J.-P. and Ekwall, K. (2015). The Fun30 chromatin remodeler Fft3 controls nuclear organization and chromatin structure of insulators and subtelomeres in fission yeast. *PLoS Genet.* 11, e1005101 10.1371/journal.pgen.1005101 25798942 PMC4370569

[JCS251769C46] Strauss, B., Harrison, A., Coelho, P. A., Yata, K., Zernicka-Goetz, M. and Pines, J. (2018). Cyclin B1 is essential for mitosis in mouse embryos, and its nuclear export sets the time for mitosis. *J. Cell Biol.* 217, 179-193. 10.1083/jcb.201612147 29074707 PMC5748970

[JCS251769C47] Sveiczer, A., Novak, B. and Mitchison, J. M. (1996). The size control of fission yeast revisited. *J. Cell. Sci.* 109, 2947-2957. 9013342 10.1242/jcs.109.12.2947

[JCS251769C48] Takemoto, A., Kawashima, S. A., Li, J.-J., Jeffery, L., Yamatsugu, K., Elemento, O. and Nurse, P. (2016). Nuclear envelope expansion is crucial for proper chromosomal segregation during a closed mitosis. *J. Cell Sci.* 129, 1250-1259. 10.1242/jcs.181560 26869222 PMC4813296

[JCS251769C49] Win, T. Z., Stevenson, A. L. and Wang, S.-W. (2006). Fission yeast Cid12 has dual functions in chromosome segregation and checkpoint control. *Mol. Cell. Biol.* 26, 4435-4447. 10.1128/MCB.02205-05 16738311 PMC1489130

[JCS251769C50] Wood, E. and Nurse, P. (2015). Sizing up to divide: mitotic cell-size control in fission yeast. *Annu. Rev. Cell Dev. Biol.* 31, 11-29. 10.1146/annurev-cellbio-100814-125601 26566110

[JCS251769C51] Woods, A., Sherwin, T., Sasse, R., MacRae, T. H., Baines, A. J. and Gull, K. (1989). Definition of individual components within the cytoskeleton of Trypanosoma brucei by a library of monoclonal antibodies. *J. Cell Sci.* 93, 491-500. 2606940 10.1242/jcs.93.3.491

