# Supplementary material for: Identification of mutants with increased variation in cell size at onset of mitosis in fission yeast
Source: J Cell Sci. 2021 Feb 11;134(3):jcs251769. doi: 10.1242/jcs.251769 (PMC7888708; doi:10.1242/jcs.251769)
Supplement: Supplementary information [file joces-134-251769-s1.pdf]

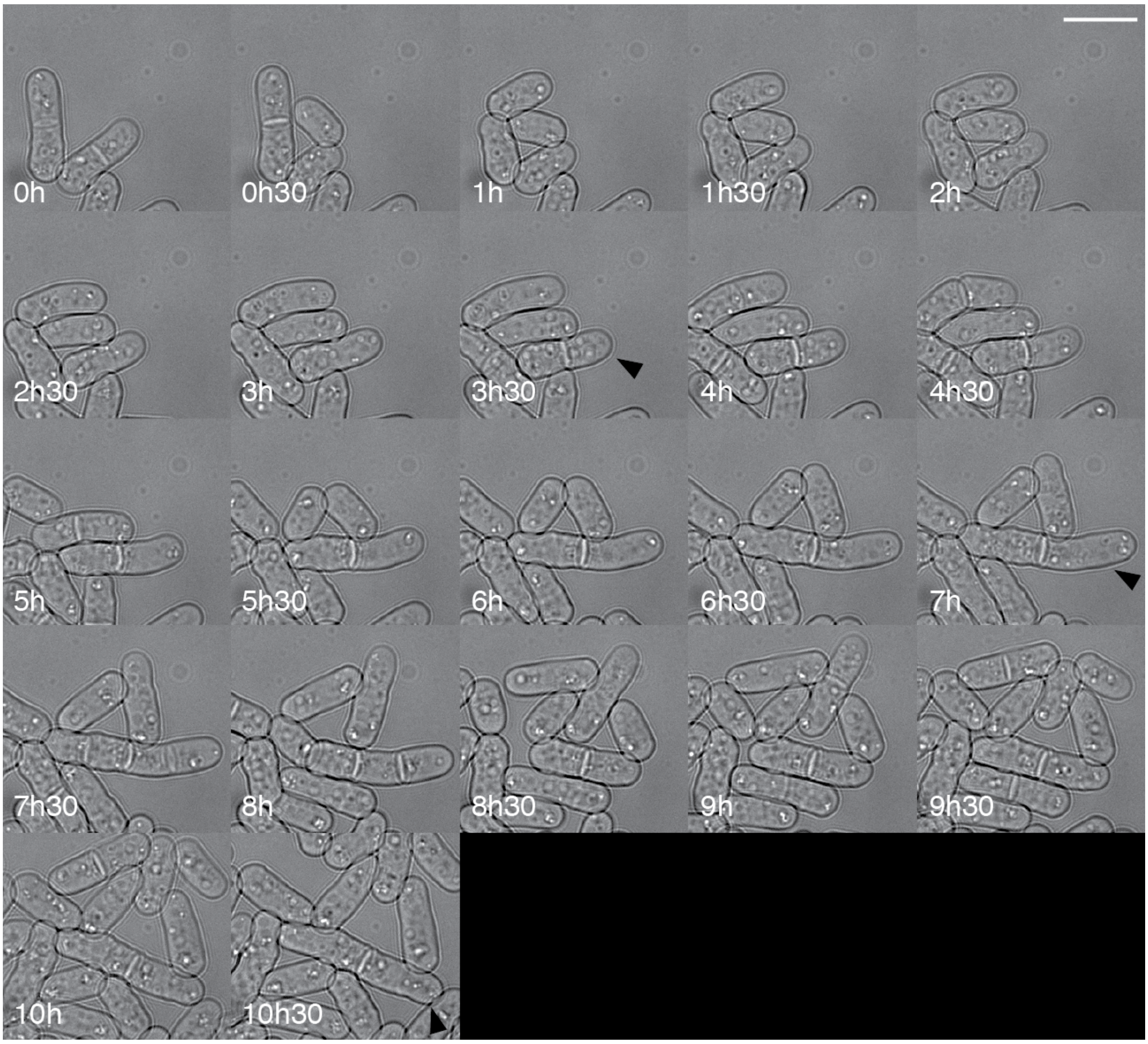

Fig. S1. *mms19Δ* causes cells to delay in septation. Stills taken from a brightfield time-lapse movie of *mms19Δ* cells growing in a microfluidic chamber. Times indicate the time lapsed from the start of imaging, which was 1 hour after loading the cells into the microfluid chamber. Cells grown at 32 °C in YE4S. Scale bar represents 10 μm.

Journal of Cell Science • Supplementary information

Table S1. Strains used in this study.		
**Strain**	**Genotype**	**Origin**
PN1	*972 h-*	Lab collection
LW166	*dph1Δ::KanMX6 ade6-M216 ura4-D18 leu1-32 h+*	Kim et al., 2010
LW167	*pek1Δ::KanMX6 ade6-M210 ura4-D18 leu1-32 h+*	Kim et al., 2010
LW168	*mms19Δ::KanMX6 ade6-M216 ura4-D18 leu1-32 h+*	Kim et al., 2010
LW169	*moe1Δ::KanMX6 ade6-M216 ura4-D18 leu1-32 h+*	Kim et al., 2010
LW170	*mga2Δ::KanMX6 ade6-M216 ura4-D18 leu1-32 h+*	Kim et al., 2010
LW171	*SPAC869.03cΔKanMX6 ade6-M210 ura4-D18 leu1-32 h+*	Kim et al., 2010
LW172	*cid12ΔKanMX6 ade6-M216 ura4-D18 leu1-32 h+*	Kim et al., 2010
LW173	*aps1Δ::KanMX6 ade6-M216 ura4-D18 leu1-32 h+*	Kim et al., 2010
LW174	*atd1Δ::KanMX6 ade6-M216 ura4-D18 leu1-32 h+*	Kim et al., 2010
LW175	*sft1Δ::KanMX6 ade6-M216 ura4-D18 leu1-32 h+*	Kim et al., 2010
LW176	*csn2Δ::KanMX6 ade6-M216 ura4-D18 leu1-32 h+*	Kim et al., 2010
LW177	*dad3Δ::KanMX6 ade6-M216 ura4-D18 leu1-32 h+*	Kim et al., 2010
LW178	*hpt1Δ::KanMX6 ade6-M216 ura4-D18 leu1-32 h+*	Kim et al., 2010
LW179	*med20Δ::KanMX6 ade6-M216 ura4-D18 leu1-32 h+*	Kim et al., 2010
LW181	*SPBC557.02cΔ::KanMX6 ade6-M216 ura4-D18 leu1-32 h+*	Kim et al., 2010
LW183	*trm112Δ::KanMX6 ade6-M216 ura4-D18 leu1-32 h+*	Kim et al., 2010
LW216	*cid12Δ::KanMX6 h-*	This study
LW228	*mga2Δ::KanMX6 h-*	This study
FN391	*cdc11-119 elo2-mCherry::NatR leu1-32 ura4-D18 h+*	Lab collection
LW311	*cid12Δ::KanMX6 cdc11-119 elo2-mCherry::NatR leu1? ura4? h?*	This study
LW314	*mga2Δ::KanMX6 cdc11-119 elo2-mCherry::NatR leu1? ura4? h?*	This study
FR1205	*wee1Δ::kanMX6 h+*	Navarro and Nurse, 2012
FR735	*leu1Δ::Pcdc13::cdc13-L-cdc2(T14A Y15F)as::cdc13 3'UTR::ura4+ cdc2Δ::ScLEU2 cdc13Δ::NatMX6 ura4-D18 h-*	Navarro and Nurse, 2012
PN5013	*leu1Δ::Pcdc13::cdc13-L-cdc2(as)::cdc13 3'UTR::ura4+ cdc2Δ::ScLEU2 cdc13Δ::NatMX6 h+*	Lab collection
LW188	*mga2Δ::KanMX6 leu1Δ::Pcdc13::cdc13-L-cdc2(T14A Y15F)as::cdc13 3'UTR::ura4+ ura4-D18 cdc2Δ::ScLEU2 cdc13Δ::NatMX6 h?*	This study
LW194	*mga2Δ::KanMX6 leu1Δ::Pcdc13::cdc13-L-cdc2as::cdc13 3'UTR::ura4+ ura4-D18 cdc2Δ::ScLEU2 cdc13Δ::NatMX6 h?*	This study
LW321	*wee1Δ::hph leu1Δ::Pcdc13::cdc13-L-cdc2::cdc13 3'UTR::ura4+ cdc2Δ::KanMX6 cdc13Δ::NatMX6 cig1Δ::ura4+ cig2Δ::ura4+ puc1Δ::ura4+ ura4-D18 h?*	This study
LW334	*wee1Δ::hph leu1Δ::Pcdc13::cdc13-L-cdc2(T14A Y15F)::cdc13 3'UTR::ura4+ cdc2Δ::KanMX6 cdc13Δ::NatMX6 cig1Δ::ura4+ cig2Δ::ura4+ puc1Δ::ura4+ ura4-D18 h?*	This study
MBY1818	*cdr2Δ::ura4+ ura4-D18 leu1-32 h-*	Bimbó et al, 2005
UKK2198-1B	*cdr2Δ::ura4+ mga2Δ::KanMX6 ura4-D18 leu1-32 h?*	This study
UKK1151-4C	*nem1Δ::KanMX6 cut11:GFP:ura4+ ura4-D18 leu1-32 h+*	This study
UKK2140-1B	*nem1Δ::KanMX6 mga2Δ::KanR cut11:GFP:ura4+ ura4-D18 leu1-32 h?*	This study
22	*leu1-32 h-*	Lab collection

Journal of Cell Science • Supplementary information

UKK2094-7C	*mga2Δ::KanMX6 leu1-32 ade6 h?*	This study
FY7287	*cdc2-1w leu1-32 h-*	Lab collection
FY8156	*cdc2-3w h-*	Lab collection
UKK2885-1C	*cdc2-1w mga2Δ::KanMX6 leu1-32 h-*	This study
UKK2874-1A	*cdc2-3w mga2Δ::KanMX6 leu1-32 ade6? h-*	This study
UKK2886-2A	*elo2-mCherry::NatR cdc13:GFP:ScLEU2 leu1-32 ura4-D18 ade6 h?*	This study
UKK2887-2A	*mga2Δ::KanMX6 cut11-mCherry::NatR cdc13:GFP:ScLEU2 leu1-32 ura4-D18 ade6 h?*	Lab collection
UKK2886-3D	*cdc11-119 elo2-mCherry::NatR cdc13:GFP:ScLEU2 leu1-32 ura4-D18 ade6 h?*	This study
UKK2887-3D	*mga2Δ::KanMX6 cdc11-119 cut11-mCherry::NatR cdc13:GFP:ScLEU2 leu1-32 ura4-D18 ade6 h?*	This study

Reference

Bimbó, A., Jia, Y., Poh, S. L., Karuturi, R. K., den Elzen, N., Peng, X., Zheng, L., O'Connell, M., Liu, E. T., Balasubramanian, M. K. and Liu, J. (2005). Systematic deletion analysis of fission yeast protein kinases. *Eukaryot. Cell* **4**, 799-813. doi: 10.1128/EC.4.4.799-813.2005.

Journal of Cell Science • Supplementary information
